# The Impact of Clinical Success Levels on Postoperative RNFL Development After Trabeculectomy

**DOI:** 10.3390/vision10030057

**Published:** 2026-08-20

**Authors:** Caroline Bormann, Carlo Fiore, Xiao Shang, Nathanael Urs Häner, Martin S. Zinkernagel, Jan Darius Unterlauft

**Affiliations:** 1 University Eye Hospital, University of Leipzig, Liebigstrasse 10-14, 04103 Leipzig, Germany; 2 Department of Ophthalmology, Inselspital, Bern University Hospital, University of Bern, 3010 Bern, Switzerland; carlo.fiore@insel.ch (C.F.); xiao.shang@students.unibe.ch (X.S.); nathanael.haener@insel.ch (N.U.H.); martin.zinkernagel@insel.ch (M.S.Z.)

**Keywords:** trabeculectomy, RNFL thickness, clinical success level, primary open-angle glaucoma

## Abstract

Background: Trabeculectomy is the standard surgical treatment for advanced glaucoma, yet the relationship between postoperative intraocular pressure (IOP) and long-term structural preservation remains unclear. We investigated longitudinal changes in peripapillary retinal nerve fiber layer (RNFL) thickness following trabeculectomy in primary open-angle glaucoma and evaluated whether lower postoperative IOP levels are associated with improved structural stability. Methods: This retrospective consecutive case series included 106 eyes undergoing trabeculectomy between 2010 and 2020 with follow-up of up to 5 years. RNFL thickness was assessed using spectral-domain optical coherence tomography at baseline and during follow-up. IOP, glaucoma medication use, best-corrected visual acuity, and visual field mean defect were recorded longitudinally. Results: Mean IOP decreased from 23.8 ± 0.9 mmHg preoperatively to 10.5 ± 0.7 mmHg at 1 month and remained significantly reduced at 5 years (13.8 ± 0.7 mmHg), with sustained reduction in medication use. RNFL thickness declined significantly from 64.4 ± 2.0 µm during the first postoperative year to 57.2 ± 1.6 µm and subsequently remained relatively stable. While eyes achieving a postoperative IOP <12 mmHg showed no statistically significant RNFL thinning compared to baseline, eyes with higher postoperative IOP exhibited greater structural decline despite meeting conventional success criteria. Conclusions: Trabeculectomy provides durable IOP reduction; however, early structural loss may still occur. Our findings suggest that lower postoperative IOP levels may be associated with greater structural preservation, although this relationship requires further investigation.

## 1. Introduction

Glaucoma comprises a group of chronic, progressive optic neuropathies characterized by irreversible loss of retinal ganglion cells (RGCs) and their axons, resulting in corresponding visual field defects and, in advanced stages, permanent visual impairment or blindness [1,2]. Elevated intraocular pressure (IOP) is the most important, and only modifiable, risk factor; however, the mechanisms linking IOP elevation to progressive neurodegeneration remain incompletely understood. To date, IOP reduction is the only intervention proven to slow or halt disease progression [3].

Trabeculectomy (TE), first described by Cairns and Watson in the 1960s, remains the gold standard surgical procedure for achieving substantial and sustained IOP reduction [4,5,6]. By facilitating aqueous humor drainage from the anterior chamber into the sub-Tenon’s and subconjunctival space, trabeculectomy can effectively lower even markedly elevated IOP levels [7]. Postoperative disease control is typically assessed by functional testing using standard automated perimetry (SAP) and structural evaluation of peripapillary retinal nerve fiber layer (RNFL) thickness measured by spectral domain optical coherence tomography (SD-OCT), a quantitative surrogate for RGC axonal integrity.

Definitions of surgical success after trabeculectomy are primarily based on postoperative IOP levels and the need for adjunctive medication. In contrast, postoperative structural changes in RGC-containing retinal layers have received comparatively less attention. Recent evidence, including our previous analyses, suggests that RNFL thinning may continue for several months after trabeculectomy despite adequate IOP reduction [8,9].

The present retrospective study therefore aimed to characterize longitudinal RNFL changes up to five years after trabeculectomy in POAG eyes and to evaluate their association with predefined surgical success criteria based on IOP reduction and medication requirements at three years postoperatively. A better understanding of postoperative structural dynamics may contribute to a more comprehensive interpretation of surgical outcomes beyond conventional pressure-based definitions.

## 2. Materials and Methods

This retrospective consecutive case series included patients with primary open-angle glaucoma (POAG) who underwent trabeculectomy at the Department of Ophthalmology, Inselspital, Bern University Hospital, Switzerland, between January 2010 and December 2020. All eligible cases within the study period were considered for inclusion. Indications for surgery comprised uncontrolled intraocular pressure (IOP) or documented structural and/or functional progression—defined as increasing visual field defects and/or progressive retinal nerve fiber layer (RNFL) thinning—despite maximally tolerated topical and/or systemic IOP-lowering therapy, as well as contraindications or intolerance to medical treatment. A postoperative control visit at approximately 3 years was considered a particularly important follow-up time point; however, this was not achievable for all patients, resulting in a variable number of eyes available for analysis at each postoperative time point.

The study adhered to the tenets of the Declaration of Helsinki. Ethical approval was obtained from the Ethics Committee of the Canton of Bern (BASEC-ID: 2022-01046; final approval 15 March 2023). Due to the retrospective nature of the study, informed consent was waived in accordance with local regulations.

All trabeculectomies were performed using a standardized fornix-based conjunctival approach. Mitomycin C (0.2 mg/mL; compounded in-house by the Hospital Pharmacy, Inselspital, Bern University Hospital, Bern, Switzerland) was applied to the scleral bed for 2 min and thoroughly rinsed with balanced salt solution. A 4 × 4 mm^2^ scleral flap was created, followed by entry into the anterior chamber and peripheral iridectomy. The scleral flap was secured with 2–4 non-absorbable 10-0 sutures (Nylon 10-0 (Ethilon^®^, Ethicon Inc./Johnson & Johnson, Somerville, NJ, USA) depending on intraoperative aqueous humor outflow. The conjunctiva was closed at the limbus using 3–4 absorbable 10-0 interrupted sutures (Vicryl 10-0 (Polyglactin 910, Ethicon Inc./Johnson & Johnson, Somerville, NJ, USA) and one non-absorbable mattress suture (Nylon 10-0 (Ethilon^®^, Ethicon Inc./Johnson & Johnson, Somerville, NJ, USA) to ensure watertight wound closure.

Postoperative follow-up examinations were routinely scheduled at days 1 and 2; weeks 1–4; and months 3, 6, 12, 24, 36, and 60. Postoperative topical therapy consisted of tobramycin (Tobrex^®^, Novartis Pharma Schweiz AG, Risch, Switzerland) four times daily for 2 weeks, atropine 0.5% (Atropine SDU Faure^®^, Théa Pharma S.A., Clermont-Ferrand, France) twice daily for 1 week and prednisolone acetate (Ultracortenol^®^, Agepha Pharma s.r.o., Zug, Switzerland) four times daily for 2 weeks followed by weekly tapering. Additional bleb-modulating interventions, including needling, 5-fluorouracil (5-FU; 0.1 mL, 50 mg/mL: compounded in-house by the Hospital Pharmacy, Inselspital, Bern University Hospital, Bern, Switzerland) injections, and laser suture lysis, were performed as clinically indicated based on slit-lamp assessment of bleb morphology, typically within the first 3 postoperative months.

Baseline demographic variables (age, sex, laterality) were extracted from electronic medical records. IOP was measured using Goldmann applanation tonometry (Haag-Streit, Köniz, Switzerland) at baseline and at each follow-up visit. The number of IOP-lowering medications and best-corrected visual acuity (BCVA) were documented at the same time points. BCVA was assessed using Snellen charts and converted to logarithm of the minimum angle of resolution (logMAR) units for statistical analysis. Peripapillary RNFL thickness was measured using spectral-domain optical coherence tomography (SD-OCT; Spectralis, Heidelberg Engineering, Heidelberg, Germany) at baseline and at 6 and 12 months as well as 2, 3, and 5 years postoperatively. Automated manufacturer-provided segmentation software was used for RNFL measurements (Heidelberg Eye Explorer [HEYEX 2], Heidelberg Engineering, Heidelberg, Germany). Standard automated perimetry (Octopus 600s, Haag-Streit, Köniz, Switzerland) using a 24-2 strategy was performed at 6 months and at 1, 2, 3, and 5 years after surgery.

The primary outcome measure was longitudinal change in peripapillary RNFL thickness. Secondary outcomes included IOP reduction, number of IOP-lowering medications, and surgical success at 3 years postoperatively. The main classes of antiglaucoma medications include prostaglandin analogs, beta-blockers, alpha-2 adrenergic agonists, carbonic anhydrase inhibitors, Rho kinase inhibitors, and fixed-dose combination therapies. Surgical success required a ≥20% reduction of IOP compared to baseline values before trabeculectomy and an absolute IOP between 6 mmHg and predefined upper thresholds (≤21, ≤18, ≤15, or ≤12 mmHg, depending on stratification). Complete success was defined as meeting the IOP criteria without adjunctive IOP-lowering medication, whereas qualified success allowed additional medication provided that the number did not exceed the preoperative regimen.

To meet the success criteria, no additional surgical intervention was allowed during the follow-up period after trabeculectomy except laser suture lysis or needling procedures with 5-FU. All cases not meeting these criteria were considered failures.

The IOP thresholds used for stratification (<12 mmHg, 12–21 mmHg, failure) were adopted from commonly used categories in the glaucoma surgical literature.

Missing data were tolerated due to the retrospective design, and no imputation procedures were performed. Analyses were conducted using available-case data at each respective time point. Data were entered into Microsoft Excel (Version 16, Microsoft Corp., Redmond, WA, USA) and analyzed using SPSS (IBM Corp., Version 24.0, Chicago, IL, USA). Continuous variables are presented as mean ± standard error of the mean (SEM), and categorical variables as frequencies. Within-group comparisons over time were performed using the Wilcoxon signed-rank test for paired non-normally distributed data. Between-group comparisons (e.g., success versus failure groups) were conducted using the Mann–Whitney U test. Associations between categorical variables were assessed using the chi-square test.

All statistical tests were two-sided, and a *p*-value ≤ 0.05 was considered statistically significant.

## 3. Results

A total of 106 eyes of 106 patients with primary open-angle glaucoma (POAG) who underwent trabeculectomy between 2010 and 2020 were included in the analysis. Mean age at the time of surgery was 64.1 ± 1.5 years, and 53 patients (50%) were female. The right eye was operated on in 46 cases (43.4%). Ten eyes (9.5%) had undergone prior glaucoma surgery (deep sclerectomy), and two eyes had received cyclophotocoagulation before trabeculectomy. Further demographic and baseline characteristics are summarized in Table 1.

### 3.1. IOP and IOP-Lowering Medication

**Figure 1:**
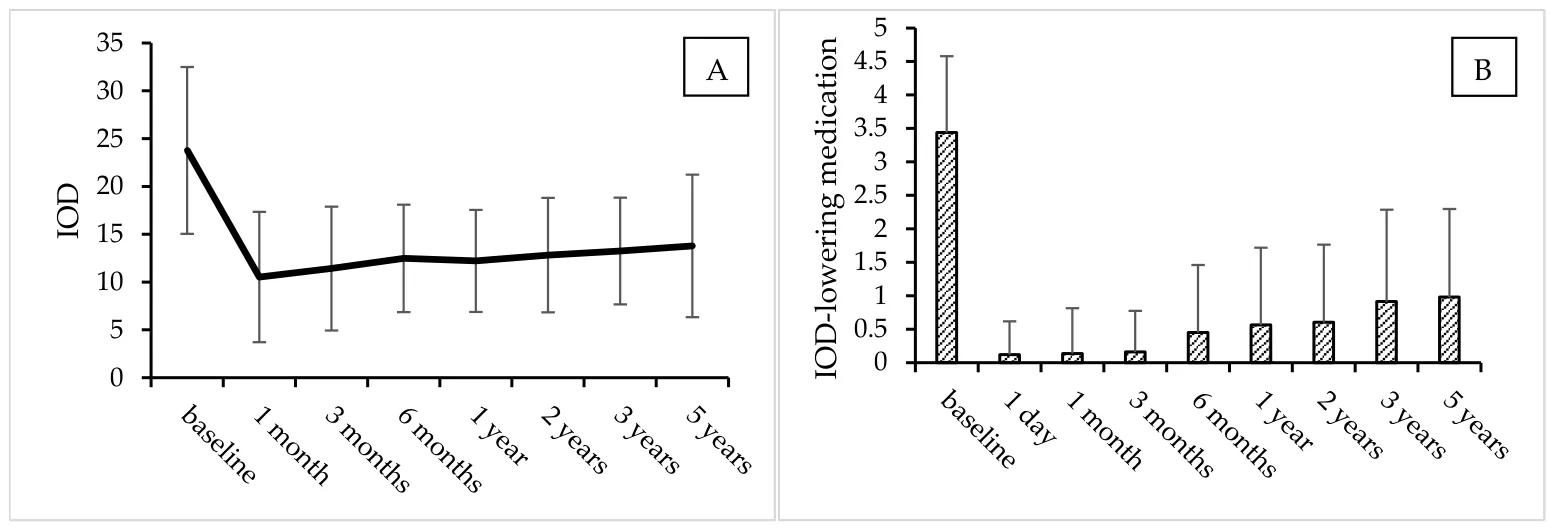
Development of IOP (**A**) and number of IOP-lowering medications (**B**) in the complete group of POAG eyes undergoing trabeculectomy.

**Figure 2:**
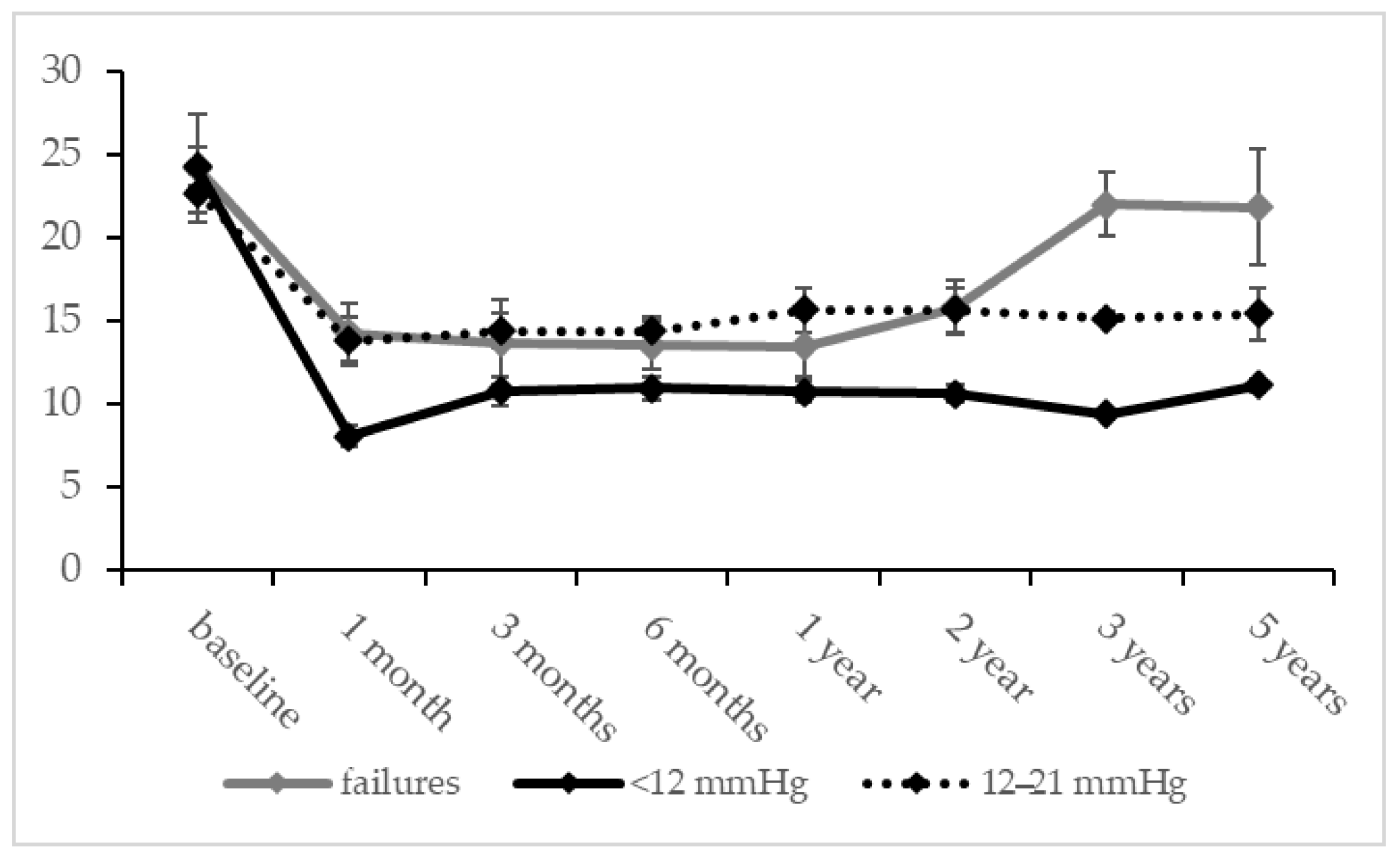
Development of intraocular pressure in the 3 groups of POAG eyes stratified for success and failure 3 years after trabeculectomy; failure *n* = 13, <12 mmHg *n* = 62, 12–21 mmHg *n* = 93.

**Figure 3:**
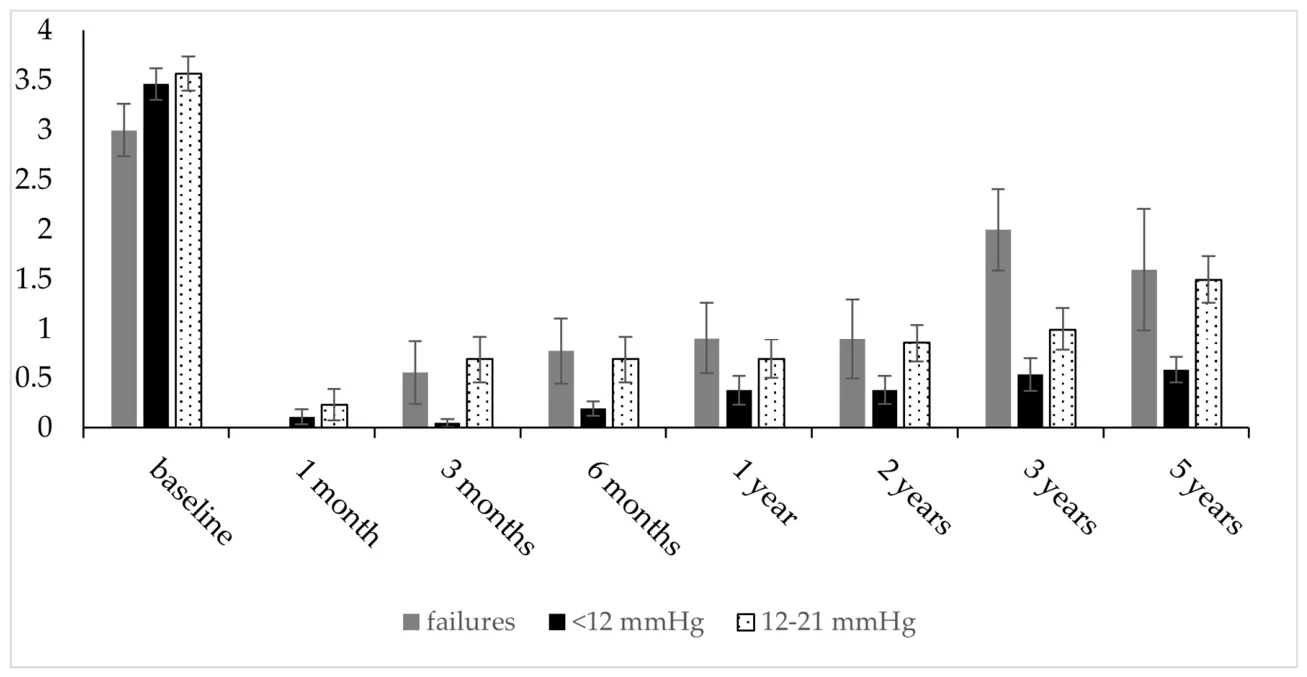
Development of the number of IOP-lowering medications in the 3 groups of eyes undergoing trabeculectomy stratified for success or failure at the follow-up 3 years after surgery; failure *n* = 13, <12 mmHg *n* = 62, 12–21 mmHg *n* = 93.

Mean preoperative IOP was 23.8 ± 0.9 mmHg. Mean IOP decreased to 10.5 ± 0.7 mmHg at 1 month postoperatively and gradually increased thereafter, reaching 13.8 ± 0.7 mmHg at 5 years. Compared with baseline, IOP was significantly reduced at all postoperative follow-up visits (all *p* ≤ 0.05; Table 2, Figure 1). The absolute number of IOP-lowering medications followed a similar pattern. At baseline, patients used a mean of 3.4 ± 0.1 medications. This decreased to 0.1 ± 0.1 at 1 month postoperatively and increased gradually to 0.6 ± 0.1 at 1 and 2 years, and 1.0 ± 0.1 at 5 years. The reduction in medication use remained statistically significant compared with baseline throughout the 5-year follow-up period (all *p* ≤ 0.05; Table 2, Figure 1). The exact development of IOP and number of IOP-lowering agents in the three groups stratified for success and failure at the follow-up visit three years after surgery is shown in Table 3 as well as in Figure 2 and Figure 3.

### 3.2. Visual Acuity, Visual Fields

According to the Hodapp-Parrish-Anderson classification based on visual field mean deviation, 20% of our patients presented with early-stage glaucoma, 43% with moderate-stage glaucoma, and 37% with advanced-stage glaucoma.

Best-corrected visual acuity (BCVA) remained stable over the 5-year follow-up period, apart from a transient perioperative decrease at day 1 and week 1 after surgery. Comparisons between baseline and follow-up measurements at 3 and 6 months, and at 1, 2, 3, and 5 years showed no statistically significant differences (Table 3). Similarly, visual field mean defect (MD) remained stable during follow-up. Mean MD was 11.5 ± 0.7 dB at baseline and 11.6 ± 0.7 dB at 5 years. No statistically significant differences were observed between baseline and follow-up examinations at 1, 2, 3, or 5 years (Table 3). Additionally, correlation analysis between RNFL thickness and visual field mean deviation did not reach statistical significance.

### 3.3. RNFL Thickness

**Figure 4:**
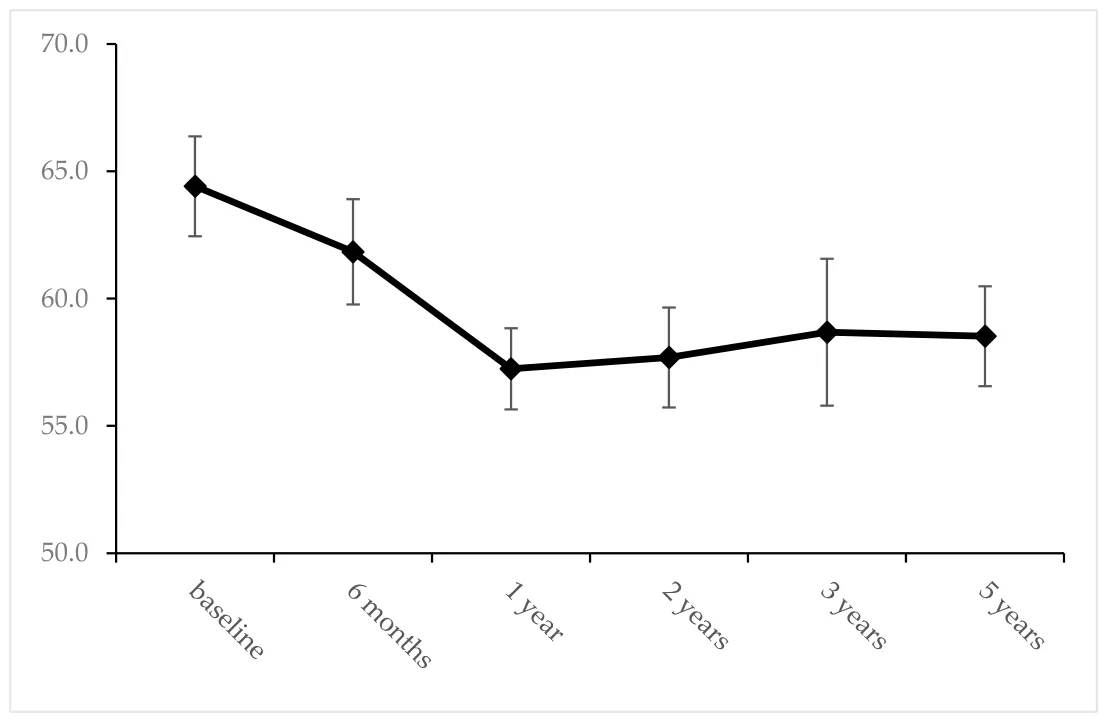
Development of mean RNFL thickness during 5 years of follow-up after trabeculectomy in the complete group of eyes analyzed.

In the complete cohort of eyes, mean peripapillary RNFL thickness decreased from 64.4 ± 2.0 µm at baseline to 61.8 ± 2.1 µm at 6 months and 57.3 ± 1.6 µm at 1 year postoperatively (Figure 4). Thereafter, RNFL thickness remained relatively stable, measuring 58.5 ± 2.0 µm at 5 years. Compared with baseline values, RNFL thickness was significantly reduced from 6 months onward at all postoperative time points compared to baseline (all *p* ≤ 0.05; Table 2 and Table 4). The largest decrease occurred between baseline and 6 months postoperatively. Comparisons between subsequent follow-up visits did not demonstrate further statistically significant changes (Table 4).

### 3.4. Clinical Success Level

**Figure 5:**
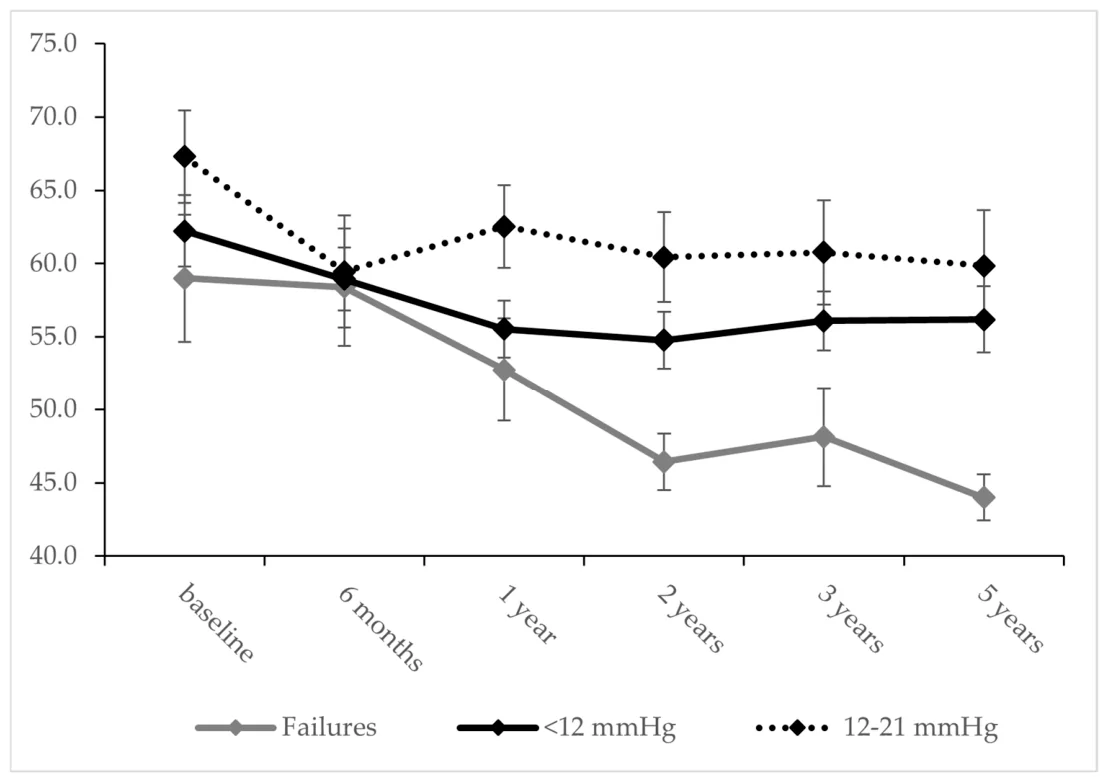
Development of mean RNFL thickness during 5 years of follow-up after trabeculectomy in the 3 groups of eyes stratified for success or failure 3 years after surgery; failure n = 9, <12 mmHg n = 30, 12–21 mmHg n = 67.

At 3 years postoperatively, surgical success (complete and qualified combined) was achieved in 93 eyes (87.7%) using an IOP threshold of ≤21 mmHg and in 62 eyes (58.5%) using a threshold of ≤12 mmHg. Due to the small resulting group sizes for final IOP values of ≤21, ≤18, and ≤15 mmHg at three years after trabeculectomy, these 3 groups were combined into one (i.e., IOP ≤ 21 mmHg) for further statistical analysis. In eyes achieving success with a postoperative IOP ≤ 12 mmHg, mean RNFL thickness was 62.2 ± 2.4 µm at baseline, 55.5 ± 1.9 µm at 1 year, and 56.2 ± 2.3 µm at 5 years (Table 4, Figure 5). In this subgroup, comparisons between baseline and follow-up RNFL measurements did not reach statistical significance. In 67 eyes achieving success with a postoperative IOP ≤ 21 mmHg but >12 mmHg, mean RNFL thickness decreased from 67.3 ± 3.2 µm at baseline to 62.5 ± 2.8 µm at 1 year and 59.8 ± 3.8 µm at 5 years. In this group, RNFL thickness was significantly reduced compared with baseline from 6 months onward (Table 4, Figure 5). In the 13 eyes (12.2%) not meeting predefined surgical success criteria at 3 years, mean RNFL thickness decreased from 59.0 ± 4.3 µm at baseline to 52.8 ± 3.5 µm at 1 year and 44.0 ± 1.6 µm at 5 years. In this subgroup, RNFL thickness was significantly lower than baseline from 6 months onward at all follow-up examinations (Table 4, Figure 5).

### 3.5. Postoperative Complications

Postoperative complications occurred in a minority of eyes. Early complications included hypotony in 8 eyes (7.5%), hyphema in 4 eyes (3.8%), choroidal effusion in 4 eyes (3.8%), wound leak in 4 eyes (3.8%), corneal erosions in 4 eyes (3.8%), shallow anterior chamber in 4 eyes (3.8%), and aqueous misdirection in 1 eye (0.9%). Hypotony maculopathy occurred in 3 eyes (2.8%). Late complications were rare, with cystoid macular edema in 2 eyes (1.9%), and hypotony and wound leak each in 1 eye (0.9%).

## 4. Discussion

In this retrospective consecutive case series of 106 eyes with POAG followed for up to five years after trabeculectomy, we confirmed the sustained efficacy of trabeculectomy in significantly reducing intraocular pressure (IOP) and medication burden. Despite effective pressure control, a significant decline in peripapillary RNFL thickness was observed during the first postoperative year in the overall cohort, while visual field indices remained stable throughout follow-up. Importantly, stratification by surgical success demonstrated that postoperative IOP levels were associated with the magnitude of structural change: eyes achieving IOP levels < 12 mmHg at three years did not exhibit statistically significant RNFL thinning compared with baseline, whereas eyes with higher postoperative IOP—despite meeting conventional success criteria—showed continued structural decline.

The IOP-lowering efficacy of trabeculectomy observed in our cohort is consistent with previously published multicenter data. Edmunds et al. reported a reduction in mean IOP from 26.2 mmHg to 14.4 mmHg one year after surgery in more than 1200 cases, with approximately two-thirds achieving a ≥33% IOP reduction [10]. Similarly, Kirwan et al. demonstrated a decrease in mean IOP from 23.0 ± 5.5 mmHg to 12.4 ± 4.0 mmHg and a substantial reduction in medication burden two years postoperatively in over 400 eyes [11]. A recent 11-year follow-up of a randomized trial similarly reported sustained complete and qualified success rates of trabeculectomy over the long term, further supporting its durability as a first-line surgical option [12]. Our results align with these findings and confirm the long-term pressure-lowering effect of trabeculectomy under routine clinical conditions.

At three years postoperatively, 87.7% of eyes met predefined surgical success criteria at an IOP threshold of <21 mmHg, and 58.5% achieved IOP levels < 12 mmHg. The corresponding proportions of eyes meeting each IOP threshold at all follow-up visits are shown in Figure S1. Structural outcomes differed between these subgroups. In eyes with postoperative IOP < 12 mmHg, RNFL thickness remained statistically unchanged compared with baseline throughout the five-year follow-up period. In contrast, eyes with postoperative IOP ≥ 12 mmHg demonstrated significant RNFL thinning from six months after surgery onwards, despite fulfilling conventional success definitions. The subgroup not meeting success criteria showed the most pronounced structural decline.

The discrepancy between progressive RNFL thinning and stable mean deviation (MD) in our cohort is consistent with the established structure-function relationship in glaucoma. This is supported by OCT data showing that other structural parameters remain useful for assessing visual function even after RNFL thickness has reached its measurement floor [13]. Changes in RNFL thickness often precede the development of glaucomatous visual field loss, reflecting the sequence in which structural ganglion cell loss typically precedes measurable perimetric change [14]. Furthermore, structure-function correlations are known to vary markedly among individuals, so that at a given level of RNFL thickness the degree of visual field loss can differ substantially, complicating the interpretation of isolated MD values as a progression marker [15].

These findings suggest that conventional pressure-based definitions of surgical success may not fully capture postoperative structural stability. While earlier landmark studies such as the Advanced Glaucoma Intervention Study (AGIS) demonstrated visual field stability in eyes with IOP consistently below 18 mmHg, advances in OCT technology now allow detection of structural changes that may precede measurable functional deterioration [16].

In our cohort, RNFL thinning during the first postoperative year occurred in the absence of significant changes in visual field mean defect, underscoring the possibility of structural progression without concurrent functional decline. Taken together, these observations support combining structural and functional parameters when assessing postoperative disease activity, since functional testing alone—and pressure control alone—may underestimate true progression, particularly in advanced-stage eyes [12,16].

Postoperative RNFL thinning following trabeculectomy has been described previously. Chua et al. reported a mean RNFL loss of approximately 4 µm during the first postoperative year, and Demirtas et al. observed significant RNFL reduction within the first year after surgery, without further significant decline in subsequent years [8,17]. Our results are consistent with these observations, demonstrating that the most pronounced RNFL reduction occurred within the first six months after surgery, followed by relative stabilization thereafter. A recent multicenter study of 462 eyes undergoing trabeculectomy, deep sclerectomy, Xen microstent, or PreserFlo microshunt similarly reported a decline in peripapillary RNFL thickness during the first postoperative year without further significant decline thereafter and identified greater baseline RNFL thickness and higher preoperative IOP as independent predictors of postoperative RNFL thinning [18]. Similarly, structural reversal of disc cupping following IOP reduction has been demonstrated after PRESERFLO microshunt implantation, suggesting that biomechanical changes at the optic nerve head in response to rapid IOP lowering are not specific to trabeculectomy but represent a broader phenomenon across IOP-lowering glaucoma surgeries [19].

One possible explanation for the early RNFL loss may be attributable to cell dysfunction induced by oxidative stress caused by higher preoperative intraocular pressure. Whether this early decline reflects continued glaucomatous damage, delayed structural response to prior injury, measurement variability, or biomechanical changes at the optic nerve head following rapid IOP reduction remains to be clarified.

In addition, the floor effect should also be taken into account when analyzing RNFL thickness. This refers to the fact that once RNFL thickness reaches a value of <50 µm, additional axonal loss cannot be reliably quantified by OCT. In our study, 23% of patients had a preoperative global RNFL thickness of <50 µm prior to trabeculectomy. To account for this limitation, we repeated the analysis using only patients without a floor effect (RNFL ≥ 50 µm) and found the same pattern of disease progression as in the complete cohort.

From a clinical perspective, our findings support the concept that lower postoperative IOP targets may be associated with improved structural stability. Although causality cannot be inferred from this retrospective design, the absence of significant RNFL thinning in eyes achieving IOP < 12 mmHg suggests that more stringent pressure control may confer structural benefit beyond conventional success thresholds. These observations emphasize the importance of integrating structural imaging into postoperative follow-up and not relying solely on IOP measurements or visual field testing when evaluating surgical outcomes.

Several limitations must be acknowledged. The retrospective single-center design introduces potential selection and information bias. Missing data were tolerated without imputation, which may have influenced longitudinal estimates. The sample size of certain subgroups was limited, particularly in eyes not meeting success criteria. Furthermore, OCT-based RNFL measurements may be influenced by segmentation errors or floor effects in advanced disease, potentially affecting longitudinal interpretation.

A further limitation relates to the long observation period, with baseline measurements in 2010, shortly after spectral-domain OCT became commercially available in 2007. Over this time, OCT segmentation algorithms and software were repeatedly updated, and quality-control criteria such as minimum signal strength were less standardized in earlier years. This could affect the absolute RNFL thickness values independent of true biological change. Scan decentration and variability in operator experience with image acquisition may have further contributed to measurement variability, particularly early in the study period. While all scans were reviewed for adequate quality, we cannot fully exclude an influence of evolving device technology and user experience.

Additionally, postoperative biochemical changes, physiological retinal nerve fiber layer loss, and the limited reproducibility of OCT scans may further reduce the interpretation of longitudinal RNFL measurements. Finally, causality between achieved IOP levels and structural stabilization cannot be established.

Furthermore, the stratification by postoperative IOP at three years (<12 mmHg, 12–21 mmHg, failure) carries a substantial risk of selection bias and confounding, as patients achieving low postoperative IOP may differ systematically in baseline disease severity, glaucoma aggressiveness, or surgical response, precluding causal inference from this observational design.

In conclusion, trabeculectomy provided sustained IOP reduction and medication sparing over five years. However, significant RNFL thinning occurred predominantly during the first postoperative year, despite stable visual field indices. Eyes achieving postoperative IOP levels below 12 mmHg demonstrated structural stability, whereas higher IOP levels—even within conventional success definitions—were associated with continued RNFL decline. These findings suggest that pressure-based definitions of surgical success may not fully reflect structural outcomes and highlight the importance of comprehensive postoperative monitoring incorporating both functional and structural assessments.

## 5. Conclusions

Trabeculectomy provides durable IOP reduction but may not uniformly prevent early structural loss. Our findings suggest that lower postoperative IOP levels may be associated with improved structural preservation.

## Figures and Tables

**Table 1:** Baseline demographic data for eyes undergoing trabeculectomy, which were included for the current analysis.

number of eyes	106
mean age; years	64.1 ± 1.5
laterality; right:left	46:60
Gender; female:male	53:53
baseline mean IOP; mmHg	23.8 ± 0.9
baseline mean number of IOP-lowering medication; n	3.4 ± 0.1
baseline mean visual acuity; logMAR	0.4 ± 0.1
baseline mean RNFL thickness; µm	64.4 ± 2.0

**Table 2:** Results for IOP, number of IOP-lowering medications, BCVA, SAP and RNFL thickness collected from 106 eyes during the 5 years of post-surgical follow-up. IOP: intraocular pressure; BCVA: best-corrected visual acuity; SAP: standard automated perimetry; RNFL: retinal nerve fiber layer.

	Baseline	1 Month	3 Months	6 Months	1 Year	2 Years	3 Years	5 Years
	*n* = 106	*n* = 95	*n* = 81	*n* = 75	*n* = 77	*n* = 74	*n* = 86	*n* = 80
IOP [mmHg]	23.8 ± 0.9	10.5 ± 0.7	11.4 ± 0.6	12.5 ± 0.6	12.2 ± 0.5	12.8 ± 0.6	13.3 ± 0.6	13.8 ± 0.7
comparison to baseline (Wilcoxon-test), *p*=	n.a.	<0.001	<0.001	<0.001	<0.001	<0.001	<0.001	<0.001
IOP-loweringmedication [n]	3.4 ± 0.1	0.1 ± 0.1	0.2 ± 0.1	0.5 ± 0.1	0.6 ± 0.1	0.6 ± 0.1	0.9 ± 0.1	1.0 ± 0.1
comparison to baseline (Wilcoxon-test), *p*=	n.a.	<0.001	<0.001	<0.001	<0.001	<0.001	<0.001	<0.001
BCVA [logMAR]	0.4 ± 0.1	0.5 ± 0.1	0.4 ± 0.1	0.3 ± 0.1	0.4 ± 0.1	0.4 ± 0.1	0.4 ± 0.1	0.5 ± 0.1
comparison to baseline (Wilcoxon-test), *p*=	n.a.	<0.001	0.611	0.487	0.250	0.797	0.204	0.635
MD of SAP [dB]	11.5 ± 0.7	-	-	11.1 ± 0.7	12.3 ± 0.7	11.5 ± 0.6	12.0 ± 0.7	11.6 ± 0.7
comparison to baseline (Wilcoxon-test), *p*=	n.a.	n.a.	n.a.	0.569	0.648	0.136	0.236	0.236
mean RNFL thickness [µm]	64.4 ± 2.0	-	-	61.8 ± 2.1	57.2 ± 1.6	57.7 ± 2.0	58.7 ± 2.9	58.5 ± 2.0
comparison to baseline (Wilcoxon-test), *p*=	n.a.	n.a.	n.a.	0.003	<0.001	<0.001	<0.001	<0.001

**Table 3:** Results for IOP and number of IOP-lowering medications developed during follow-up of 5 years in the three subgroups of eyes stratified for surgical success at the follow-up visit 3 years after surgery.

	Groups	Baseline	1 Month	3 Months	6 Months	1 Year	2 Years	3 Years	5 Years
IOP [mmHg]	<12 mmHg	24.3 ± 1.2	8.1 ± 0.6	10.8 ± 0.9	10.9 ± 0.7	10.7 ± 0.6	10.6 ± 0.5	9.4 ± 0.3	11.1 ± 0.4
	12–21 mmHg	22.7 ± 1.2	13.8 ± 1.5	14.4 ± 1.0	14.4 ± 0.9	15.7 ± 1.3	15.7 ± 1.3	15.2 ± 0.4	15.4 ± 1.6
	failures	24.2 ± 2.2	14.3 ± 1.8	13.7 ± 2.6	13.5 ± 1.5	13.5 ± 1.8	15.8 ± 1.6	22.0 ± 1.9	21.8 ± 3.5
IOP-lowering medication [n]	<12 mmHg	3.5 ± 0.2	0.1 ± 0.1	0.1 ± 0.1	0.2 ± 0.1	0.4 ± 0.2	0.4 ± 0.1	0.5 ± 0.2	0.6 ± 0.1
	12–21 mmHg	3.6 ± 0.2	0.2 ± 0.2	0.7 ± 0.2	0.7 ± 0.2	0.7 ± 0.2	0.9 ± 0.2	1.0 ± 0.2	1.5 ± 0.2
	failures	3.0 ± 0.3	0 ± 0	0.6 ± 0.3	0.8 ± 0.3	0.9 ± 0.4	0.9 ± 0.4	2.0 ± 0.4	1.6 ± 0.6

**Table 4:** Results for mean RNFL thickness in the different groups of success and different resulting IOP and failures after trabeculectomy.

	Complete Group	Comparison to Baseline (Wilcoxon-Test), *p*=	Group < 12 mmHg	Comparison to Baseline (Wilcoxon-Test), *p*=	Group 12–21 mmHg	Comparison to Baseline (Wilcoxon-Test), *p*=	Failures	Comparison to Baseline (Wilcoxon-Test), *p*=
baseline	64.4 ± 2.0	n.a.	62.2 ± 2.4	n.a.	67.3 ± 3.2	n.a.	59.0 ± 4.3	n.a.
6 months	61.8 ± 2.1	0.003	58.9 ± 2.2	0.569	59.5 ± 3.8	0.008	58.4 ± 4.0	0.027
1 year	57.2 ± 1.6	<0.001	55.5 ± 1.9	0.648	62.5 ± 2.8	0.018	52.8 ± 3.5	0.034
2 years	57.7 ± 2.0	<0.001	54.8 ± 2.0	0.136	60.4 ± 3.1	0.006	46.4 ± 1.9	0.043
3 years	58.7 ± 2.9	<0.001	56.1 ± 2.0	0.236	60.8 ± 3.6	0.006	48.1 ± 3.3	0.035
5 years	58.5 ± 2.0	<0.001	56.2 ± 2.3	0.236	59.9 ± 3.8	0.020	44.0 ± 1.6	0.015

## Data Availability

The original contributions presented in this study are included in the article/Supplementary Material. Further inquiries can be directed to the corresponding author(s).

## Supplementary Materials

The following supporting information can be downloaded at: https://www.mdpi.com/article/10.3390/vision10030057/s1, Figure S1: Cumulative success probability.
