# Supplementary material for: The Impact of Clinical Success Levels on Postoperative RNFL Development After Trabeculectomy
Source: Vision (Basel). 2026 Aug 20;10(3):57. doi: 10.3390/vision10030057 (PMC13517604; doi:10.3390/vision10030057)
Supplement: Supplementary file 1 [file vision-10-00057-s001.zip › vision-4483466-supplementary.pdf]

Supplementary Materials: The Impact of Clinical Success Levels on Postoperative RNFL Development After Trabeculectomy

Caroline Bormann, Carlo Fiore, Xiao Shang, Nathanael Urs Häner, Martin S. Zinkernagel and Jan Darius Unterlauft

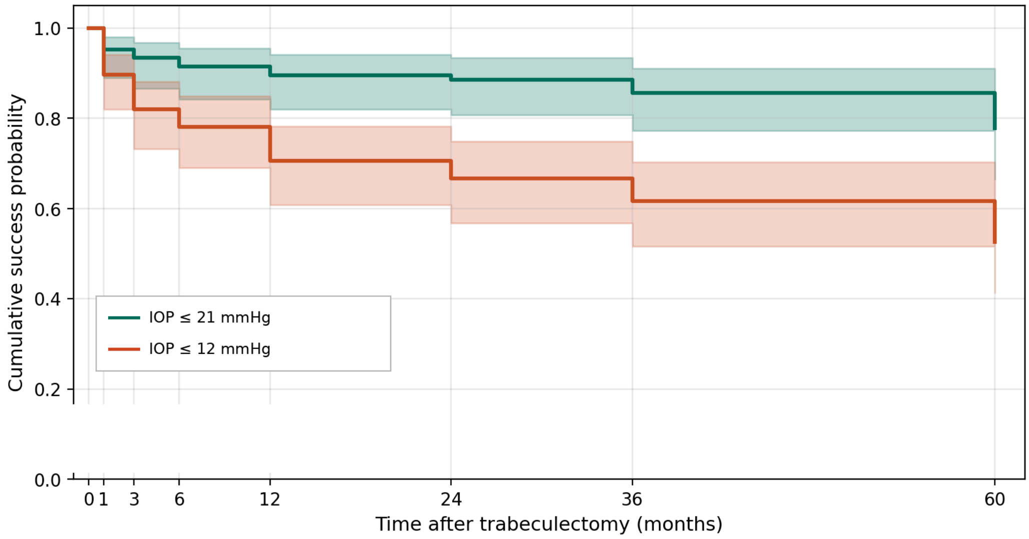

Figure S1: cumulative success probability.
